# Inequalities and age-period-cohort effects of global and regional burden for interstitial lung disease and pulmonary sarcoidosis: based on Global Burden of Disease study 2021

**DOI:** 10.3389/fmed.2025.1471402

**Published:** 2025-05-26

**Authors:** Lei Zhang, Yixian Wu, Jiarui Zhang, Junxiong Qiu, Yipu Xiao, Zhuochen Chen, Yanwei Zhang, Jingjun Han

**Affiliations:** ^1^Department of Thoracic Surgery, The Eighth Affiliated Hospital, Sun Yat-sen University, Shenzhen, China; ^2^Department of Cardiovascular Surgery, Sun Yat-sen Memorial Hospital, Sun Yat-sen University, Guangzhou, China; ^3^Department of Orthopedics, Sun Yat-sen Memorial Hospital, Sun Yat-sen University, Guangzhou, China; ^4^Department of Anesthesiology, Sun Yat-sen Memorial Hospital, Sun Yat-sen University, Guangzhou, China; ^5^Immunization Planning Institute, Center for Disease Control and Prevention, Shenzhen, China

**Keywords:** Global Burden of Disease, interstitial lung disease, pulmonary sarcoidosis, disability-adjusted life years, inequality analysis, age-period-cohort model, joinpoint analysis

## Abstract

**Background:**

Interstitial lung disease (ILD) and pulmonary sarcoidosis pose significant disease burdens on a global scale.

**Methods:**

The underlying data was derived from the Global Burden of Disease (GBD) 2021 database. The disease burden was quantified through age-standardized rates (ASRs) and numbers of disability-adjusted life years (DALYs), prevalence, and incidence. The indicators' dynamic trends are captured through Joinpoint analysis and its average annual percentage change (AAPC). Concentration Index (CI) and Slope Index (SI) were employed to characterize the imbalances in the global disease burden. The age-period-cohort model was utilized to elucidate temporal trends in sociobiological factors on the disease burden.

**Results:**

Between 1992 and 2021, the ASRs of ILD and pulmonary sarcoidosis have increased globally. For another, the imbalanced distribution of disease burden increased, with more pronounced in high socio-demographic index (SDI) regions. Besides, age-standardized DALY rates (ASDRs) for ILD and pulmonary sarcoidosis were positively associated with age and period effects worldwide and complexly associated with cohort effects, increasing between 1882 and 1928 birthed cohorts and reducing after 1982. However, the distinct period-effect and cohort-effect curves observed in Africa which illustrate a negative correlation trend are noteworthy.

**Conclusion:**

Between 1992 and 2021, the global burden of ILD and pulmonary sarcoidosis increased, which were higher in high SDI countries and among the elderly. Furthermore, the disease burden increased with age and period and decreased for those born after 1982. However, the risk ratios of disease in Africa were negative with period-effect and cohort-effects, deserving effective interventions.

## Introduction

Interstitial lung disease (ILD) is a diffuse parenchymal lung disease associated with high morbidity and mortality (1), especially in those manifesting progressive pulmonary fibrosis. According to Table 1, the total number of global prevalence due to ILD and pulmonary sarcoidosis had reached 4306630, and the age-standardized prevalence rate (ASPR) was 50.011 cases per 100,000 population in 2021. Pulmonary sarcoidosis is one of the etiological factors contributing to ILD, characterized by non-caseating granuloma formations that primarily affect the lung interstitium, leading to inflammation and fibrosis (2). Currently, the incidence of the disease is still increasing worldwide with the majority of cases occurring in middle-aged and elderly men (3). The disease presents most commonly as restrictive lung disease resulting in reduced forced vital capacity (FVC) and diffusing capacity (DLCO) (1, 4). Some epidemiological studies have suggested that interactions between smoking, genetic susceptibility, age, and male gender are important reasons for the rising incidence (5).

**Table 1:** The regional number and ASR of DALYs, incidence, and prevalence in ILD and pulmonary sarcoidosis in 2021, and their annual percentage change with 95% UI from 1992 to 2021.

**Location**	**DALYs**			**Incidence**			**Prevalence**		
	**Number**	**ASDR**	**AAPC**	**Number**	**ASIR**	**AAPC**	**Number**	**ASPR**	**AAPC**
	**2021**	**2021**	**1992–2021**	**2021**	**2021**	**1992–2021**	**2021**	**2021**	**1992–2021**
**Global**	4,042,150 (3,489,790–4,516,880)	47.618 (41.258–53.165)	2.005 (1.907–2.102)	390,267 (346,393–433,403)	4.545 (4.055–5.038)	1.708 (1.651–1.766)	4,306,630 (3,802,950–4,898,710)	50.011 (44.239–56.774)	1.446 (1.407–1.486)
**Sex**									
**Male**	2,237,270 (1,839,500–2,555,200)	57.788 (47.499–65.767)	1.936 (1.811–2.061)	214,681 (190,533–238,498)	5.365 (4.802–5.953)	1.730 (1.661–1.800)	2,149,200 (1,902,460–2,433,400)	53.725 (47.690–60.590)	1.525 (1.476–1.573)
**Female**	1,804,880 (1,465,710–2,216,380)	39.486 (31.952–48.620)	2.086 (1.953–2.219)	175,586 (155,725–195,607)	3.886 (3.456–4.313)	1.687 (1.648–1.725)	2,157,430 (1,902,020–2,464,270)	47.301 (41.740–54.008)	1.367 (1.332–1.401)
**Five world regions**									
**Africa**	184,688 (98,853–290,733)	25.541 (13.796–40.777)	−0.124 (−0.168–−0.080)	15,186 (13,236–17,436)	1.941 (1.713–2.172)	0.284 (0.265–0.304)	165,016 (140,126–194,957)	21.352 (18.410–24.770)	0.582 (0.555–0.608)
**America**	1,062,010 (973,189–1,132,060)	80.028 (73.614–85.268)	2.624 (2.281–2.969)	106,426 (94,969–117,771)	8.043 (7.226–8.854)	2.114 (2.065–2.164)	1,129,600 (1,005,550–1,268,330)	85.062 (76.127–95.382)	1.540 (1.450–1.631)
**Asia**	2,149,900 (1,717,920–2,607,940)	43.501 (35.196–52.632)	2.110 (1.937–2.283)	214,774 (188,728–241,485)	4.212 (3.715–4.709)	1.923 (1.885–1.961)	2,349,940 (2,049,170–2,711,400)	46.263 (53.091–40.401)	1.889 (1.851–1.927)
**Europe**	638,548 (584,836–682,111)	40.709 (37.713–43.480)	2.449 (2.005–2.896)	53,412 (48,547–58,413)	3.766 (3.429–4.122)	1.853 (1.809–1.898)	656,432 (591,234–735,102)	45.321 (40.505–51.027)	1.410 (1.348–1.472)
**Oceania**	8,024 (5,581–12,403)	74.630 (50.209–118.430)	0.554 (0.445–0.663)	419 (385–456)	4.200 (3.906–4.509)	0.780 (0.761–0.799)	4,880 (4,391–5,411)	49.164 (44.658–54.301)	0.935 (0.914–0.956)

DALYs, Disability-adjusted life-years rate; ILD, Interstitial lung disease; ASDR, Age-standardized disability-adjusted life-years rate; ASMR, Age-standardized mortality rate; AAPC, Average annual percentage Change, 95% uncertain intervals are displayed in the brackets.

Although the COVID-19 epidemic has renewed worldwide attention to the disease (6), there is still a significant gap in the study of its epidemiological model in regions with different SDI levels (7, 8). Currently, high sociodemographic index (SDI) regions bear the greatest disease burden of ILD and sarcoidosis due to their higher screening and consultation rates, but the influence of demographic and temporal factors (age factors, period factors, etc. (9)) on the disease burden in these regions remains to be elucidated. Therefore, analyzing the interactions between biological factors and disease burden is crucial for developing region-specific disease control policies (10).

To further investigate and address the aforementioned questions, our study examined the impact and the trend of sociobiological factors on the disease burden of ILD and sarcoidosis in different regions. This was achieved through an overall trend analysis and multivariate decomposition epidemiological analysis using the Global Burden of Disease (GBD) 2021 database. SDI were employed respond to the economic development of the localities (7), and corresponding epidemiological models were constructed to elucidate the impact and dynamics of economic and sociodemographic factors on the burden of disease (11).

Due to the existing problems mentioned above and the increasing burden of ILD and pulmonary sarcoidosis, our study aimed to address: (i) Trends of the burden of ILD and pulmonary sarcoidosis [represented by disability-adjusted life years (DALYs)] with various SDI, ages, periods, and birth cohorts (12) in different regions between 1992 and 2021; (ii) The reasons behind trends in the burden of diseases with SDI, age, period and cohort effect and potential interventions.

## Methods

### Definition

In GBD 2021, ILD and pulmonary sarcoidosis under the Chronic Respiratory Diseases (CRD) catalog, based on the International Classification of Diseases (ICD) 10th (assigned codes D86-D86.2, D86.9, J84-J84.9) and 9th (assigned codes 135–135.9, 515, 516–516.9) editions (11). The ICD classification is the authoritative classification of diseases recognized by most countries in the world. ILD refers to a group of lung diseases affecting the interstitium (the tissue and space around the air sacs of the lungs), which is associated with diseases that affect a number of organs and tissues, or affects the body as a whole (13). Pulmonary sarcoidosis is a multisystem disorder of unknown cause characterized by the formation of immune granulomas in the lungs (14).

### Data source

Our fundamental data was derived from GBD 2021, through which we calculated indicators such as DALYs and average annual percentage change (AAPC) for ILD and pulmonary sarcoidosis. Data tables and visualizations can be accessed at https://vizhub.healthdata.org/gbd-results. This database continuously collects statistical data on diseases across various regions, genders, and age groups from 1992 to 2021, with revisions and data updated from GBD 2019. In this study, we divided the data into regions based on 204 countries and 5 continents, and further stratified them by age, period, gender, and economic level. We calculated key indicators for each stratum, including age-standardized incidence rates (ASIR), ASPRs, age-standardized DALY rates (ASDRs), and their 95% uncertainty interval (95% UI). DALYs consist of years of life lost (YLL) due to premature death and years lived with disability (YLD) caused by disease, covering both the loss of life years due to mortality and the loss of healthy years under the weight of disability. It is a representative indicator for measuring disease burden (15) and is the primary metric used and analyzed in this study.

### Inequality analysis

To ascertain the correlation between the financial degree of regions and the burden of disease, we calculated the Concentration Indices (CIs) and Slope Indices (SIs) for the age-standardized rates (ASRs) of interstitial lung disease and lung nodules from 1992 to 2021 and performed the corresponding curve visualizations. The ASRs in our study are derived from the GBD 2021 database, which applies uniform case definitions and systematic modeling across countries to mitigate the bias introduced by disparities in diagnostic capabilities. Specifically, the DisMod-MR 2.1 Bayesian meta-regression model incorporates relevant covariates, including healthcare access and quality indices, to adjust estimates in data-sparse or low-diagnosis-rate settings. CI is a measure of relative inequality, quantified as twice the area between the concentration curves and the line of equality, reflecting inequality in health relative to the economic level. We used the covariance to calculate the CI with represents: *C* = 2*cov*(*x, h*) presents *cov*(*x, h*) denotes the covariance between relative rank *x* and health *h* with μ representing the mean level of health. On the other hand, SI serves as a measure of absolute distributional inequality, indicating the slope of the regression line that depicts the relationship between the health of a given class and its relative rank within the socio-economic distribution (16). The SDI is a composite indicator of the development status of a country or region, which is assessed by combining data on the overall fertility rate of females under 25 years of age, the average level of education of females aged 15 years and older, and income per capita.

### Joinpoint regression analysis

The temporal trends in the burden of disease were examined by Joinpoint regression analysis which was effective in analyzing and presenting trends by region and period. The Bayesian Information Criterion (BIC) is used to determine the optimal number of connection points, which is implemented in the connection point regression program (version 5.0.2) developed by the National Cancer Institute (NCI) in the United States. To assess the statistical significance of temporal trend changes, we employed the Monte Carlo permutation test with a significance threshold of *p* < 0.05, as recommended by the NCI guidelines. By combining the above two methods, the fitting ability and simplicity of the Joinpoint regression analysis model are balanced. Additionally, annual percentage change (APC) and AAPC values were introduced to summarize the overall changes, with the statistical significance of changes in APC and AAPC assessed by calculating *p-*values with a threshold value of *p* < 0.05 (12).

### Age-period-cohort model

To further investigate the impact of sociobiological factors on disease burden, we applied the age-period-cohort model to analyze regional trends in age, period, and birth cohort effects in isolation (17). The age-period-cohort model was constructed, analyzed, and visualized based on the web tool: https://analysistools.nci.nih.gov/apc/help.html. This tool applies constrained generalized linear models to estimate age, period, and cohort effects by applying recognizability constraints, such as setting reference groups and comparing differences between adjacent groups. This allows for stable estimation of net drift, local drift, and relative risk. The age effect grouping was from 5 years of age, in groups of 5 years; the period effect grouping was based on 1992 as the reference point, with a 5-year cycle; the birth cohort effect took into account partially overlapping cohorts, with the 1882 birth cohort as the starting point, 1977 as the baseline reference point, and the cohort-specific to reference cohort ratio as the vertical coordinate.

### Statistical analysis

There are uncertainty intervals in this GBD study, including inconsistencies in data sources, modeling errors, and missing data. To enhance the precision and reliability of the data, we quantified the uncertainty intervals and calculated 95% UI (comprising the values at the 2.5% and 97.5% levels of the data samples), which had to be statistically significant (*p* < 0.05) (18). Except for the parts indicated, the analysis and visualization of this study were performed using the APC modeling tool, R (V.4.3.1; R Foundation for Statistical Computing), R Studio (2023.12.1; Posit, PBC), Stata (V.18; StataCorp LLC), and Joinpoint software (V.5.0.2; National Cancer Institute, USA).

## Results

### DALYs, incidence, and prevalence

**Figure 1:**
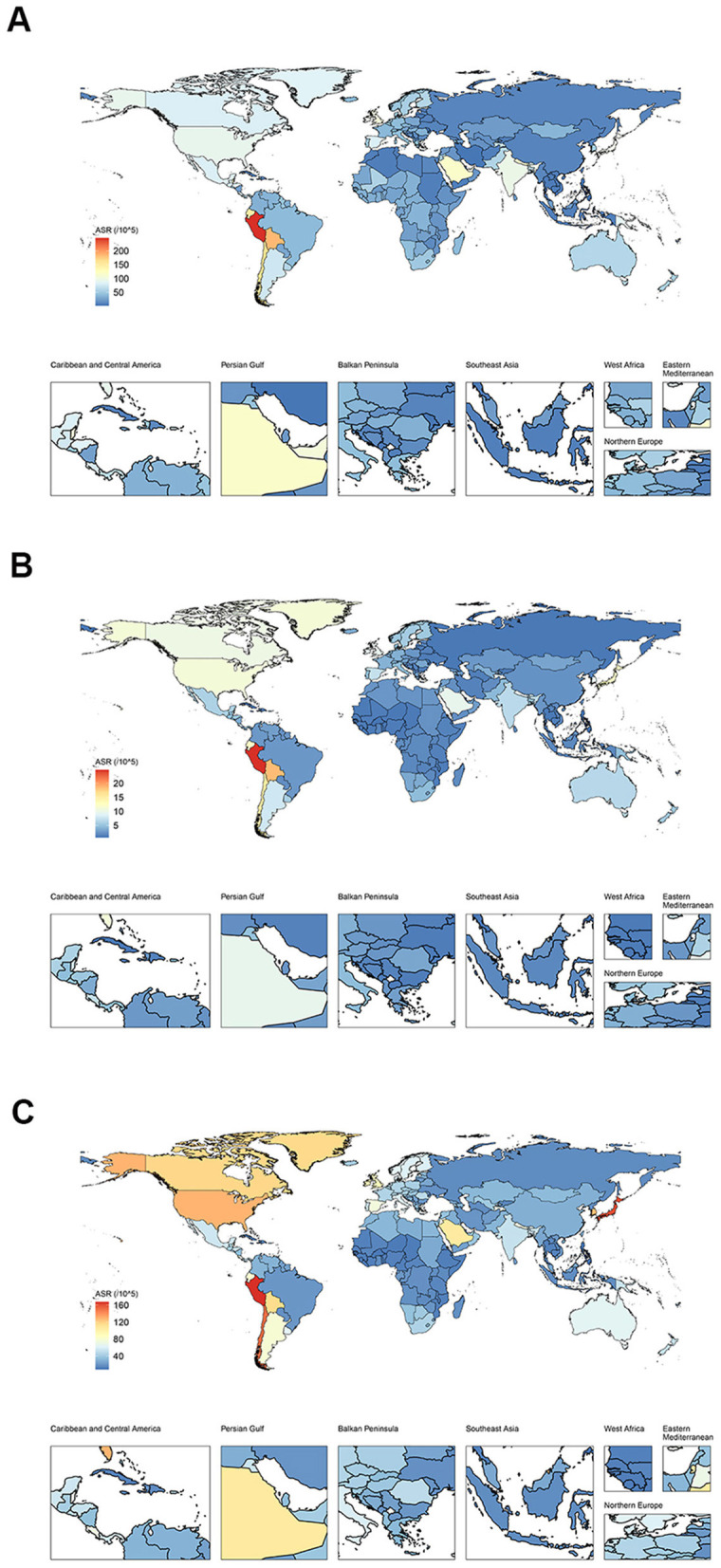
The distribution of burden in ILD and pulmonary sarcoidosis for both sexes in 2021 for 204 nations. **(A)** Global distribution of ASDR of ILD and pulmonary sarcoidosis; **(B)** Global distribution of ASIR of ILD and pulmonary sarcoidosis; **(C)** Global distribution of ASPR of ILD and pulmonary sarcoidosis. ASDR, Age-standardized disability-adjusted life-years rate; ASIR, Age-standardized incidence rate; ASPR, Age-standardized prevalence rate; ILD, Interstitial lung disease.

As of 2021, As demonstrated in Table 1, the collective number of DALYs afflicted with ILD and pulmonary sarcoidosis had reached 4,042,150 cases (95% UI: 3,489,790–4,516,880) while the ASDR had risen to 47.62 cases per 100,000 population (95% UI:41.26–53.16) globally. For another, the ASDR in males (57.79 cases per 100,000 population, 95% UI: 47.50–65.77) was 1.46 times higher than the ASDR in females (39.49 cases per 100,000 population, 95% UI: 31.95–48.62). In Figure 1A, the ASDRs of these diseases were more significant in America and Western Europe. For another, the number of individuals worldwide reached 4,306,630 cases (95% UI: 3,802,950–4,898,710). The difference in the prevalence of the disease between males (2,149,200 cases, 95% UI: 1,902,460–2,433,400) and females (2,157,430 cases, 95% UI: 1,902,020–2,464,270) is not statistically significant. As illustrated in Table 1 and Figure 1C, the highest ASPRs were observed in America (85.06 cases per 100,000 population, 95% UI: 95.38–76.13), reaching a rate nearly four times that of Africa (21.35 cases per 100,000 population, 95% UI: 18.41–24.78). At the national level, according to Supplementary Table S1 and Figure 1B, Peru owned the highest ASIR (24.73 cases per 100,000 population, 95% UI: 23.24–26.22), the highest ASPR (167.38 cases per 100,000 population, 95% UI: 155.61–179.28) and the highest ASDR (246.21 cases per 100,000 population, 95% UI: 178.27–317.79), while the lowest ASIR(0.73 cases per 100,000 population, 95% UI: 0.61–0.85), the lowest ASPR (8.34 cases per 100,000 population, 95% UI: 6.71–10.28) and the lowest ASDR (2.07 cases per 100,000 population, 95% UI: 1.54–2.62) was in the Philippines.

**Figure 2:**
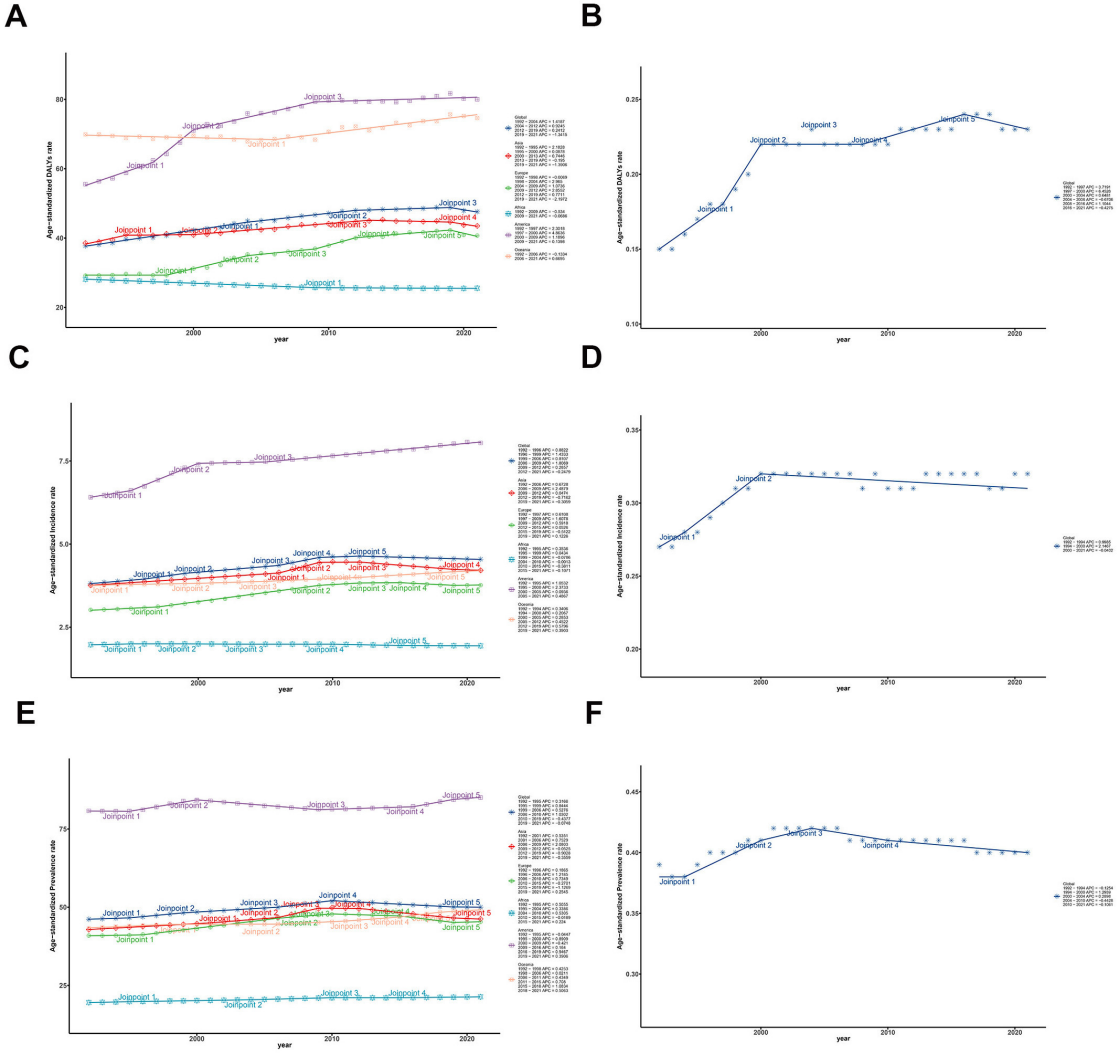
Trends of ASRs and these CIs of ILD and pulmonary sarcoidosis for both sexes in five regions from 1992 to 2021. **(A)** Trends of global and regional ASDR; **(B)** Trends of global ASDR Concentrate Index; **(C)** Trends of global and regional ASIR; **(D)** Trends of global ASIR Concentrate Index; **(E)** Trends of global and regional ASPR; **(F)** Trends of global ASPR Concentrate Index. ASDR, Age-standardized disability-adjusted life-years rate; ASIR, Age-standardized incidence rate; ASPR, Age-standardized prevalence rate; ILD, Interstitial lung disease; CI, Concentrate Index; SI, Slope Index; 95% UI, 95% Uncertain intervals.

Based on Figure 2A and Table 1, the global ASDRs for the disease have exhibited a gradual increase over the past three decades, with the AAPC of 2.01 (95% UI: 1.91–2.10). However, the Joinpoint regression analysis indicated a general downward trend in the APC of the global ASDR, with the APC dropping from 1.42 in the first 13 years (1992–2004) to −1.34 in the most recent 3 years (2019–2021). By the way, the trends of ASIR and ASPR showed limited change among five regions from 1992 to 2021 in Figures 2C, E. For another, the trends in ASDR varied across the 204 countries and five regions in Supplementary Table S1, and AAPC is lowest in Africa (−0.13, 95% UI: −0.169 to −0.08) and highest in America (2.62, 95% UI: 2.28–2.97), with Latvia having the lowest AAPC and Saint Vincent and the Grenadines highest at the national level. It is worth noting that although the DALY of Africa as a whole showed a downward trend from 1992 to 2021 (AAPC < 0), there were significant differences in the trend of DALY changes among different regions within Africa. As shown in Supplementary Table S1, South Africa's AAPC reached 0.64 (95% UI: 0.37–0.91), while some countries in sub-Saharan Africa had lower AAPCs, such as Nigeria (−1.15, 95% UI: −1.24 to −1.07), Ethiopia (−0.62, 95% UI: −0.70 to −0.54), and so on.

Collectively, despite a slight decline in the number of DALYs for ILD and pulmonary sarcoidosis globally between 1992 and 2021, there remained a significant burden of disease worldwide, especially for males and in America in 2021. Exception for Africa, global ASRs have generally been on the rise over the past three decades, with America showing the most substantial rise.

### Distribution of inequality

Over the last 30 years, the ASDRs for interstitial lung disease and pulmonary sarcoidosis have increased in most countries but decreased in some developing regions, which implies that the relationship between SDI and the burden of the disease varies between regions, contributing to the potentially unequal distribution of the two diseases. To illustrate the temporal trends of the global inequality of ILD and pulmonary sarcoidosis burden, we conducted a Joinpoint analysis of CI for ASRs and concentrate curves together with slope curves over the past 30 years for these two diseases in Figures 2B, D, F.

**Figure 3:**
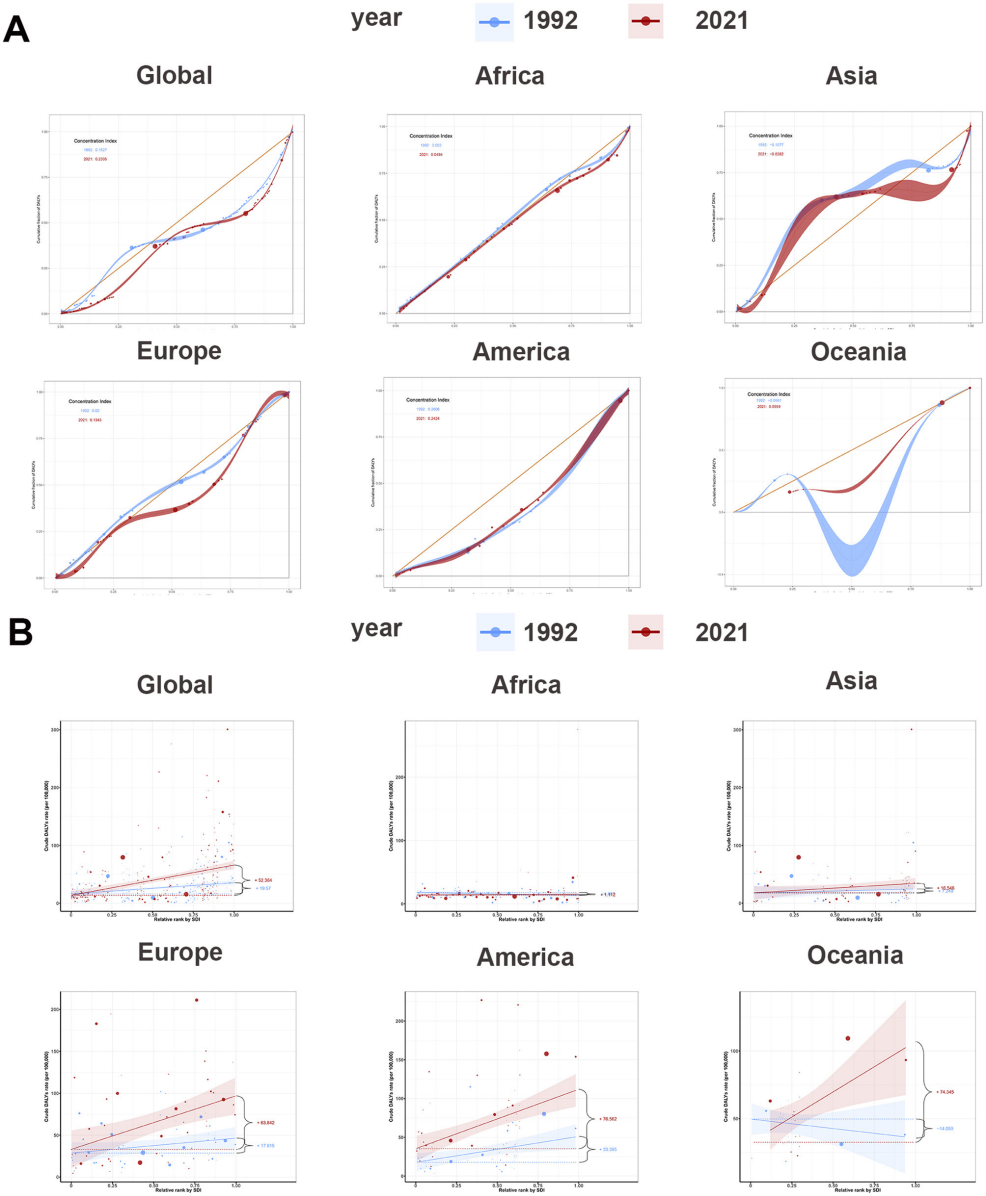
Concentrate curves and slope curves of regional ASDR of ILD and pulmonary sarcoidosis for both sexes and their CIs and SIs and their 95% UI in 1992 and 2021. **(A)** Concentrate curves of global and regional ASDR of ILD and pulmonary sarcoidosis; **(B)** Slope curves of global and regional ASDR of ILD and pulmonary sarcoidosis. ASDR, Age-standardized disability-adjusted life-years rate; ASIR, Age-standardized incidence rate; ASPR, Age-standardized prevalence rate; ILD, Interstitial lung disease; CI, Concentrate Index.

According to Figure 3A, the global concentration curves for ASDR for interstitial lung disease and lung nodules in 2021 were concentrated below the isopleths and further apart than in 1992, suggesting an uneven global disease burden of ILD and pulmonary sarcoidosis: ASDR was concentrated in high SDI countries. The global CI for ASDR increased from 0.15 (95% confidence interval: 0.09–0.22) in 1992 to 0.23 (95% confidence interval: 0.15–0.31) in 2021. As the Joinpoint analysis demonstrated in Figure 2B and Table 2, the CI trends of global ASDR included six stages, increasing from 1992 to 2004 and 2008 to 2016 and decreasing from 1997 to 2000 and 2016 to 2021. The most significant increase was from 1997 to 2000 (APC: 6.45, 95% confidence interval: 2.78–10.26), and the most significant decrease was from 2004 to 2008 (APC: −0.67, 95% confidence interval: −2.33 to 1.02). As indicated in Figure 3A and Supplementary Table S2, CIs have risen worldwide except America, with the most notable rise in Oceania (from −0.07 in 1992 to 0.10 in 2021). This implied that other factors affecting these the inequality of two diseases in Oceania, the exploration and analysis of which may assist in the screening diagnosis and prognosis of the disease.

**Table 2:** The global trends and AAPCs of CIs of ASDR, ASIR, and ASPR attributed to ILD and pulmonary sarcoidosis and their 95% confidence intervals with *p*-values from 1992 to 2021.

**Location**	**Measures**	**Trend**	**Years**	**APC/AAPC**	**Lower**	**Upper**	***p*-value**
**Global**	**ASDR**	Trend 1	1992–1997	3.719	2.811	4.636	< 0.005
		Trend 2	1997–2000	6.453	2.777	10.259	< 0.005
		Trend 3	2000–2004	0.648	−0.981	2.304	0.408
		Trend 4	2004–2008	−0.671	−2.333	1.020	0.405
		Trend 5	2008–2016	1.104	0.654	1.556	< 0.005
		Trend 6	2016–2021	−0.427	−1.169	0.320	0.238
		AAPC	1992–2021	1.513	1.017	2.012	< 0.005
	**ASIR**	Trend 1	1992–1994	0.999	−0.245	2.258	0.111
		Trend 2	1994–2000	2.147	1.869	2.425	< 0.005
		Trend 3	2000–2021	−0.040	−0.070	−0.010	0.011
		AAPC	1992–2021	0.480	0.381	0.579	< 0.005
	**ASPR**	Trend 1	1992–1994	−0.125	−0.830	0.584	0.712
		Trend 2	1994–2000	1.294	1.133	1.455	< 0.005
		Trend 3	2000–2004	0.290	−0.061	0.642	0.100
		Trend 4	2004–2010	−0.443	−0.602	−0.283	< 0.005
		Trend 5	2010–2021	−0.106	−0.154	−0.058	< 0.005
		AAPC	1992–2021	0.165	0.086	0.244	< 0.005

ASDR, Age-standardized disability-adjusted life-years rate; ASIR, Age-standardized incidence rate; ASPR, Age-standardized prevalence rate; APC, Annual percent change; AAPC, Average annual percentage Change; ILD, Interstitial lung disease; CI, Concentrate index. 95% uncertain intervals and p-values are displayed in this table.

The SI in Supplementary Table S2 and Figure 3B illustrated the overall relationship between SDI and ASDR in ILD and pulmonary sarcoidosis. Firstly, on a global scale, the ASDR for ILD and pulmonary sarcoidosis showed a positive correlation with SDI, with SI rising from 19.57 in 1992 to 52.36 in 2021. Secondly, on a continental basis, SIs had increased in five regions, with Oceania showing the most significant increase (14.06–74.35). The significant increase in SI in Oceania relative to other regions may be attributed to Australia's uneven distribution of prevention policies and medical resources between urban and rural areas, impacting early diagnosis and management of the disease.

Interestingly, between 1992 and 2021, the CI of ILD and pulmonary sarcoidosis ASDR in America slightly decreased (from 0.26 to 0.24), but SI significantly increased from 33.40 to 76.56. This implied that despite the overall narrowing of disparities in disease burden among different groups, the ASDRs for ILD and pulmonary sarcoidosis have increased more rapidly among lower socioeconomic status populations. Breakthroughs in early diagnostic and therapeutic techniques may be responsible for the overall reduction in ASDR, while environmental pollution from high industrialization in the high SDI regions of America has tilted the balance of the disease burden even further.

### Age-period-cohort effect analysis

**Figure 4:**
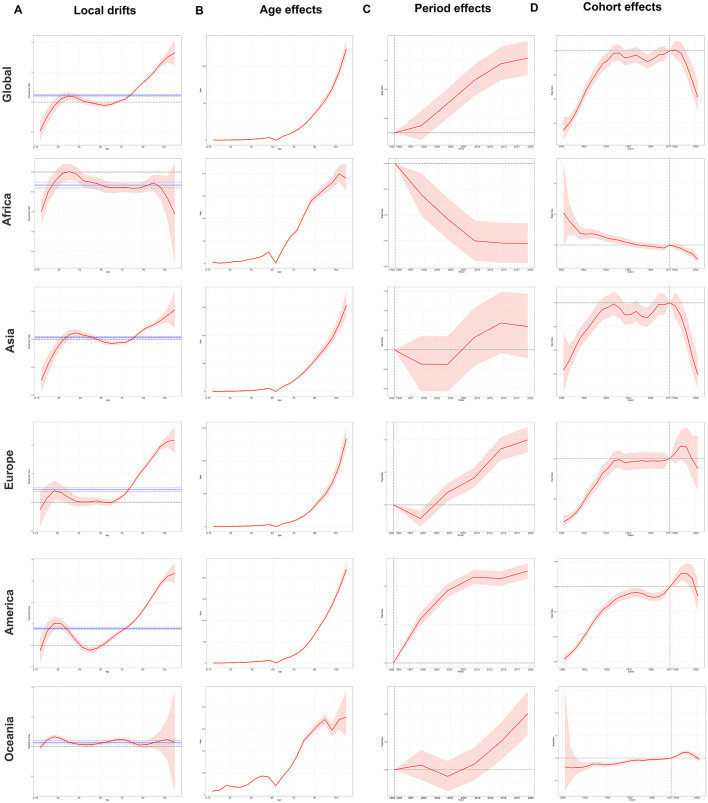
Local drifts, age, period, and cohort effect curves of global and regional DALYs of ILD and pulmonary sarcoidosis for both sexes from 1992 to 2021 and 95% UI. **(A)** Local drifts of global and regional DALYs and their net drifts; **(B)** Age effects of global and regional DALYs; **(C)** Period effects of global and regional ASDRs; **(D)** Cohort effects of global and regional DALYs. ASDR, Age-standardized disability-adjusted life-years rate; DALYs, Disability-adjusted life-years; ILD, Interstitial lung disease; 95% UI, 95% Uncertain intervals. Net drift and its 95% UI were illustrated as **solid** and **dashed lines**.

In Figure 4A, we initially evaluated and plotted local drift, which described annual trends in change over a specific age range. Subsequently, we presented net drift and its 95% UI as solid and dashed lines, respectively, and assessed the AAPC in cohort effects for each of the five regions (Figure 4A and Supplementary Table S3). First, a positive association was observed between age, period effects, and the ASDR for ILD and pulmonary sarcoidosis from 1992 to 2021 (Figures 4B, C). Second, in Figure 4D, when it comes to the cohort effects, the difference in ASDR for individuals born between 1920 and 1980 is relatively modest, with a peak observed around 1982. Besides, the local drift curves stayed above the zero line in most areas, indicating that the risk of two diseases was greater for individuals with later birth years.

The manifestation of the ASDR age effect in different regions of ILD and pulmonary sarcoidosis was broadly similar: a positive correlation in which the ASDR continued to increase with age (Figure 4B). However, it's worth noting that the period effects of the two diseases have shown an increasing trend over time in Figure 4C, except in Africa. The period effect curve in Africa continues to decline, which may be related to factors such as the predominance of inhabited populations in near-tropical rainforest areas with relatively favorable air environments. In terms of cohort effects of Figure 4D, the trends of ASDR risk in Asia, Europe, and America demonstrated a comparable pattern to the global one, exhibiting an initial increase followed by a leveling off and a subsequent decline. Differently, while the risk of ASDR in Africa continues to decline as the year of birth is delayed, the risk of ASDR in Oceania rises steadily in a more moderate trend until a downward trend begins to emerge at a turning point with the cohort born around 1990, similar to which in Europe and America. The emergence of the aforementioned phenomena may be attributed to changes in individual lifestyles and advances in medical conditions these years.

Consequently, ILD and pulmonary sarcoidosis continued to represent a significant global health concern, with increasing net drifts in ASRs observed in most continents except Africa. This indicated that the prevalence of these diseases was still on the rise. Furthermore, the problem was most severely exacerbated in Europe, which had the highest net drifts of ASDR among the five continents (Supplementary Table S3). Concurrently, the prevalence of the diseases was also significantly and positively correlated with age and peaks in the cohort born around 1985–1990. The exact cause of this peak has not yet been elucidated, but exploring its causes may help improve the early diagnosis, treatment, or prognosis of ILD and pulmonary sarcoidosis.

## Discussion

In the present investigations, we statistically evaluated the distribution of the burden of ILD and pulmonary sarcoidosis worldwide and in different regions from 1992 to 2021, as well as its relationship with SDI, age, period, and cohort effects. As of 2021, interstitial lung disease and lung nodules remained significant global health concerns, particularly affecting men and populations in America. While there has been a gradual decline in ASDRs for this disease in recent years, the uneven distribution of the disease burden is becoming more pronounced, with a tilt toward high-SDI areas. Furthermore, ILD and pulmonary sarcoidosis burden were found to be positively associated with age, period, and cohort factors in most regions. However, the period and cohort effects in Africa demonstrated divergent trends, which may informed the development of disease policies.

Our findings indicated that the global ASRs for ILD and pulmonary sarcoidosis have significantly increased in the past 30 years. The total ASDR for the disease in 2021 reached 1.60 times the 1992 total ASDR, while the ASIR and ASPR reached 1.51 and 1.43 times, respectively. The increase of ASRs in ILD and pulmonary sarcoidosis may partly stem from occupational and environmental exposures, bacterial or viral infections, the continued low rates of smoking cessation, and the aging population. Firstly, it is acknowledged that occupational and environmental exposures represent a significant risk factor. For another, long-term exposure to air pollutants (e.g., PM2.5, NOx) or hazardous substances like asbestos, silica dust, and chemical gases in certain occupations increases disease risk (19, 20). Secondly, previous studies have shown that certain bacterial, fungal, or viral infections are closely related to the incidence and prognosis of the disease (21). Furthermore, prolonged smoking can result in damage to lung tissue and chronic inflammation, which are believed to play a significant role in the development and prognosis of ILD and pulmonary sarcoidosis (22). Besides, our study identified a strong correlation between ILD and pulmonary sarcoidosis and age, with global aging significantly increasing the disease burden. Therefore, improving the living environment, taking precautions against occupational exposures, quitting smoking, and exercising appropriately to avoid premature aging of the lungs (3) may be important ways to reduce DALYs of ILD and pulmonary sarcoidosis (23).

Our study suggested that the burden of ILD and pulmonary sarcoidosis has been more pronounced in areas with high SDI over the past 30 years. Firstly, we found that the burden of the diseases tended to be higher in areas with richer healthcare resources and more advanced science and technology. Secondly, the increase in ASDR for these diseases tended to be limited in regions with lower socio-economic status (24), with Africa, dominated by countries with low to medium SDI levels, having the lowest AAPC of the five continents from 1992 to 2021. It is worth mentioning that although the AAPC of Africa as a whole has been negative over the past 30 years, DALYs in high SDI countries such as South Africa have shown an upward trend, while DALYs in low/medium SDI countries in sub Saharan Africa have mostly shown a downward trend, which is consistent with the international trend of disease burden changes and further validates our research results. Furthermore, Oceania, one of the high-SDI regions, exhibited the most pronounced increase in CI for ASDR, implying that Oceania had the most significant worsening of the disease burden imbalance. This phenomenon may be associated with several factors. On the one hand, air pollution problems due to industrialization and urbanization, and increased rates of disease diagnosis due to the development of medical technology may be responsible for the increased burden of disease in high-SDI areas (25). On the other hand, lower rates of disease diagnosis and higher proportions of infectious or nutrition-related disease burden also limited the increase in ASDR in regions with lower socioeconomic status., it is also caused by the high prevalence of smoking among males and high exposure to ambient particulate matter among females, as well as occupational exposures due to the well-developed mining, agriculture, and construction industries (26). In light of the aforementioned inferences, low-SDI areas must enhance the screening rate and diagnostic accuracy of the disease, while areas characterized by heavy industry and high urbanization should prioritize improvements in air quality, which represents a fundamental aspect of disease prevention and control (27).

To investigate the influence of sociobiological factors on the global burden of ILD and pulmonary sarcoidosis over time, we applied APC modeling. Our findings indicated that the rise in ASDR for ILD and pulmonary sarcoidosis is positively associated with age globally and with period and birth cohort in most regions. Conversely, in Africa, which is dominated by countries with medium to low SDI, the above effects are not as pronounced. From 1992 to 2021, the spread of computed tomography (CT) and its derived technologies has improved the diagnosis and accuracy of the disease (28). In addition, increased urbanization growing environmental pollution problems and occupational exposures have contributed to the disease burden, especially in countries with high SDI. Goobie et al. found a significant positive correlation between long-term exposure to PM2.5 and the risk and progression of interstitial lung disease (ILD), suggesting that air pollution particles may exacerbate lung interstitial damage through oxidative stress and inflammatory reactions (20). Statistics indicated that 90% of the African population is concentrated in land areas near rainforests and water sources, where agriculture and light industry are the main modes of production (29). Furthermore, the Tobacco Products Control Act (first enacted in 1999), led by South Africa, is considered one of the toughest anti-smoking laws in the world. These factors may partly explain the negative correlation between the period effect and the birth cohort effect in Africa. It may be posited that stricter tobacco control, optimization of industrial distribution, and environmental protection as well as pollution reversal may be important factors in reducing the disease burden.

In addition, the increase in burden of ILD and pulmonary sarcoidosis is significantly higher in individuals over the age of 63. Conversely, the Cohort Effects Study found a significant downward trend in ASDR among the birth cohort after 1982. Also in the 1980s, Electron Beam CT (EBCT) was invented in 1983 and began to be widely used in clinical practice, and the concept of World No Tobacco Day was developed and promoted in 1987. As previously stated, this phenomenon may be attributed to the dissemination of imaging technologies such as CT, the intensification of environmental pollution control, and the implementation of more rigorous anti-smoking policies (30, 31). Besides, bacterial, fungal, or viral infections were also significant factors affecting ILD and pulmonary sarcoidosis. It has been demonstrated that interstitial lung disease was more closely associated with viral infections (32), whereas the formation of lung nodules is associated with all three (33). In conclusion, quitting smoking early, implementing disease screening for individuals over 63, and avoiding infections may be effective strategies to improve or reverse the long-term increasing trend of disease burden (34, 35).

Therefore, emphasis on effective early screening for ILD and pulmonary sarcoidosis is essential, which is also evidenced by ASRs of post-1982 birth cohorts. Studies have demonstrated a more pronounced reduction in mortality from interstitial ILD and pulmonary sarcoidosis in countries that perform population-based screening, such as Russia and Kyrgyzstan, in comparison with countries that do not screen for the respective diseases, such as Iran, Peru, and Athens (36). For continents with predominantly intermediate and low SDI countries, screening policies for the disease are often inadequate and can be weighted and stratified according to population-attributable fractions (PAFs) in different geographic regions and divided into intervals to provide independent screening recommendations (37). Meanwhile, stricter tobacco control policies and more effective air quality management and improvement policies should also be implemented to control the increasing disease burden. In patients with ILD and pulmonary sarcoidosis that are believed to be caused by infectious agents, it is crucial to promptly control the infection to minimize disease progression (38, 39).

In conclusion, the prevention and treatment of ILD and pulmonary sarcoidosis necessitate a multifaceted approach. For individuals, the prevention of occupational exposure and early diagnosis and treatment, as well as the cessation of smoking at an early stage, may reduce the risk of the disease. For community groups, the promotion of health education about the disease and the organization of regular medical check-ups, such as chest X-rays, may play an important role in the tertiary prevention of the disease. At the governmental level, region-specific disease screening and prevention policies should be developed based on the pathogenesis and epidemiological regional characteristics of the disease, which may reduce the disease burden of the disease at the overall national level.

Our study expands upon prior research in several critical ways. While the earlier study by Ma et al. primarily focused on age-standardized rates of incidence, mortality, and DALYs analyzed by sex, SDI, and region from 1990 to 2019 (40), another study employed both an age-period-cohort model and a Bayesian APC (BAPC) model to project global epidemiological trends until 2030 using GBD 2019 data (10). Our study utilizes the updated GBD 2021 dataset, extending the analysis to 2021 and incorporating advanced methods such as Joinpoint analysis for AAPC, as well as CI and SI to assess global disparities in disease burden. Additionally, our application of the APC model offers deeper insights into temporal trends influenced by age, period, and cohort effects. By adopting this comprehensive approach, we provide a more nuanced understanding of the financial and demographic inequalities affecting ILD and pulmonary sarcoidosis, offering valuable insights for targeted public health strategies.

This study was subject to certain limitations. Firstly, ILD and pulmonary sarcoidosis, which were multifactorial conditions affecting primarily middle-aged and elderly individuals, frequently presented with a multitude of complications resulting from diverse causative factors. The occurrence of complications or incidental events may potentially introduce bias into the results of the analyses. The introduction of advanced statistical models such as data cleaning and pre-processing, mixed effects models, or multivariate analyses can identify and reduce relevant biases and improve research accuracy. Secondly, the GBD 2021 database did not differentiate between subtypes of interstitial lung disease and pulmonary nodules, which limited the comprehensiveness of this study by limiting the analysis of previous relationality between subtypes and different geographical areas. This limitation may be resolved with subsequent studies or database refinement. Third, the GBD database was observational, and some of the data relied on self-reporting and diagnosis in different countries and regions, which may exist problems such as missing data, differences in definitions and classifications, insufficient diagnostic accuracy, and reported correlations do not directly infer causality between the disease and related factors (41). Although the GBD database applied DisMod MR 2.1 Bayesian meta regression model to adjust data from different countries to minimize data bias caused by differences in medical technology, differences in diagnostic capabilities, especially between high SDI and low SDI regions, may still affect the true comparability of ASR. As collaboration between GBD and national authorities deepens and the process of disease diagnosis standardization continues to advance, this issue will also be addressed.

## Conclusion

ILD and pulmonary sarcoidosis represent substantial global health challenges. From 1992 to 2021, the global burden of these diseases has risen, particularly in high SDI countries and among older populations. The disease burden correlates positively with age and temporal trends, yet it declines for cohorts born after 1982. Notably, in Africa, the risk ratios for these diseases exhibited a negative trend in period and cohort effects, highlighting the need for effective interventions.

## Data Availability

The datasets presented in this study can be found in online repositories. The names of the repository/repositories and accession number(s) can be found below: https://ghdx.healthdata.org/gbd-2021.

## Supplementary material

Supplementary Table S1 The regional and national numbers and ASRs of DALY, incidence, and prevalence in ILD and pulmonary sarcoidosis in 2021, and their annual percentage change with 95% UI from 1992 to 2021. Abbreviations: DALYs, Disability-adjusted life-years rate; ILD, Interstitial lung disease; ASDR, Age-standardized disability-adjusted life-years rate; ASMR, Age-standardized mortality rate; AAPC, Average annual percentage Change, 95% uncertain intervals are displayed in the brackets.

Supplementary Table S2 The CIs and SIs of ASDR, ASIR, and ASPR attributed to ILD and pulmonary sarcoidosis for both sexes in five regions in 1992 and 2021. Abbreviations: ASDR, Age-standardized disability-adjusted life-years rate; ASIR, Age-standardized incidence rate; ASPR, Age-standardized prevalence rate; CI, Concentrate index; SI, Slope index; 95% UI, 95% Uncertain intervals; ILD, Interstitial lung disease.

Supplementary Table S3 The Net drifts and its annual percent change of DALYs, incidence, and prevalence attributed to ILD and pulmonary sarcoidosis by regions for both sexes from 1992 to 2021. Abbreviations: DALYs, Disability-adjusted life-years rate; ILD, Interstitial lung disease. 95% uncertain intervals are displayed in this table.
